# Analysis of pharmaceuticals in fish using ultrasound extraction and dispersive spe clean-up on que Z-Sep/C18 followed by LC-QToF-MS detection

**DOI:** 10.1016/j.mex.2020.101010

**Published:** 2020-07-26

**Authors:** J.M. Peña-Herrera, N. Montemurro, D. Barceló, S. Pérez

**Affiliations:** Water and Soil Quality Research Group, IDAEA-CSIC, c/Jordi Girona 18-26, 08034 Barcelona (Spain)

**Keywords:** HPLC-HRMS, SWATH, QuE Z-Sep/C18, QTOF-MS

## Abstract

The presence of pharmaceutically active compounds (PhACs) in aquatic biota has been received much less attention than their presence in surface or waste water, and it was not until the mid-2000s, this gap started to be addressed. Here, we present ***SQUEEZe*** (**S**olid-li**Q**uid **U**ltrasound **E**xtraction with Qu**E Z**-S**e**p/C18 as dispersive clean-up): a fast method for analysis of the trace 47 PhACs in fish muscle. Compared to our previously reported method [1], it offers alternatives with improvements in recoveries, number of analytes, sample volume and solvent used.

The key aspects of this method are:

• The ultrasound extraction was performed with acetonitrile/isopropanol 0.1% V/V formic acid. A clean-up step using QuE Z-Sep/C18 sorbents was employed to reduce lipid content of the extracts and further matrix effects in the detection of the analytes.
• A HPLC separation with a Kinetex EVO C18 packed column in 11 min was optimized. MS and MS/MS data were collected using SWATH acquisition on the SCIEX X500R QTOF in (+)-ESI mode.
• The method validated at 3 different concentrations levels: 5, 25 and 50 ng/g fish. It presented good intraday/interday reproducibility and absolute recoveries ≥ 60% for majority of analytes in composite homogenate muscle matrix of *Squalius cephalus*.
• 10 out 47 compounds were detected in fish samples.

## Graphical abstract

**Figure:**
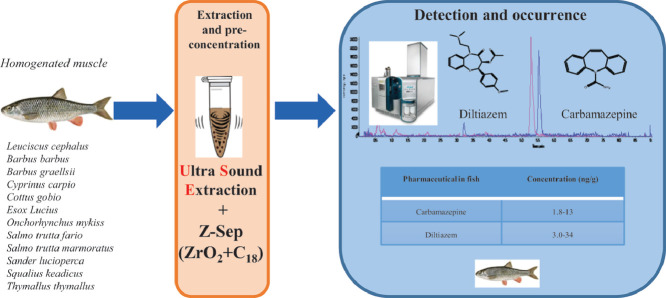

**Table:** Specifications Table

Subject Area:	Chemistry
More specific subject area:	Environmental Analytical Chemistry
Method name:	*SQUEEZe*:***S**olid-li**Q**uid **U**ltrasound **E**xtraction with Qu**E Z**-S**e**p/C18 as dispersive clean-up
Name and reference of original method:	Peña-Herrera J.M., Montemurro N., Barceló D., Pérez S.. Combining quantitative and qualitative approaches using Sequential Window Acquisition of All Theoretical Fragment-Ion methodology for the detection of pharmaceuticals and related compounds in river fish extracted using a sample miniaturized method. Journal of Chromatography A. 2020:461,009. [2]
Resource availability:	*SCIEX O.S. V.1.5 or higher*

## *Method details

Common approaches for the extraction of pharmaceutically active compounds (PhACs) from fish tissues rely on solid-liquid extraction, enzymatic microwave-assisted extraction, ultra-sound extraction (USE), QuEChERS, and pressurized liquid extraction [1,[3], [4], [5], [6], [7], [8], [9], [10]. Prior to the analysis of the extracts by LC-MS, it is critical to remove co-extracted lipids as much as possible in order to reduce matrix effects during analyte ionization in the interface. Available clean-up methods include purification by sorbents (florisil, alumina, silica gel, hydrophilic-lipophilic balance and mixed-mode cation-exchange), back-extraction of fats into highly apolar solvents (hexane or chloroform), gel permeation chromatography and freezing out [11], [12], [13], [14]. Regarding the application of sorbents, specific materials have been developed for the efficient removal of fat, which afford different strong interactions between the solid phase and the lipids. For example, dispersive Solid Phase Extraction Enhanced Matrix Removal (d-SPE EMR) acts on the principle of size exclusion and hydrophobic interactions to interact with substances that have long lipophilic chains whereas zirconium dioxide and C18 adsorbents QuE Z-Sep/C18 takes advantages of Lewis acid/base interactions [15], [16], [17]. Here, we present **S**olid-li**Q**uid **U**ltrasound **E**xtraction with Qu**E Z**-S**e**p/C18 as dispersive clean-up (***SQUEEZe***) a fast procedure to extract PhACs from fish muscle. For the sensitive and selective determination of the analytes, high resolution mass spectrometry (HRMS) on a QTOF-MS from Sciex was used employing **S**equential **W**indow **A**cquisition of All **Th**eoretical Fragment Ion **M**ass **S**pectra (SWATH) acquisition mode which is a data independent analysis-non target acquisition method, where a full scan MS event is follows by a series of all-ion-fragmentation events with narrow precursor ion ranges. This generates fragment ion spectra of lower complexity compared to those obtained upon indiscriminate fragmentation of molecular ions over a broad *m/z* range. Taken together, our proposed analytical methodology is fast, requires small sample amounts, consumes little extraction solvent, and is suitable for measuring trace levels of PhACs in fish. The selected PhACs have been chosen among the most commonly reported pharmaceutical compounds present in surface waters. These compounds can affect the aquatic biota, but are not frequently reported or analyzed in the fish matrix. However, many other contaminants are known to be present in fish samples that have not been studied or reported due to the lack of standards. Owing to the fact that SWATH acquisition acquires in parallel in scan mode, it is possible to perform a retrospective analysis into the files and retrieve analytes which were not in the target list of compounds, as reported in Peña-Herrera et al. [2].

## Sampling

Nine European chubs *(Squalius cephalus,* Linnaeus, 1758*)* sampled in 2015 from the Adige river in Italy (weight of 1 kg each, approximately) and Sava river which is a transboundary river crossing Slovenia, Croatia, Bosnia and Herzegovina and Serbia (weight of 0.2- 0.3 kg each) were selected for method validation. Moreover, 25 fish samples from four European rivers were analyzed for the applicability of the protocol. Sampling campaigns were conducted in the Adige, Sava, Evrotas (Greece), and Llobregat (Spain) rivers in 2015. From Italy, 10 samples from *Salmo trutta fario, Salmo trutta marmoratus, Thymallus thymallus, Cottus gobio, and Squalius cephalus* were examined. From Greece, four individuals from *Squalius keadicus* were analyzed. From Spain, three samples from *Cyprinus carpio* and *Barbus graellsii* were tested; and finally from the Sava River 15 samples of *Squalius cephalus, Barbus barbus, Salmo trutta fario, Onchorhynchus mykiss, Esox lucius, and Sander lucioperca* were evaluated. After sampling, the fish were transported to refrigeration boxes for laboratory analysis. The muscle tissue of each fish was initially separated from the other tissues, including epidermal tissue. All the muscles were finely homogenized separately with a lab blender and TissueLyzer sample disruptor (Quiagen, Hilden Germany) and qualitatively analyzed to determine the presence or absence of target analytes. Subsequently, 10 gs of homogenized muscle free of target analytes were taken from each fish and mixed in a pool of samples for validation purposes. The samples were stored in laboratory at −25 °C to be used for analysis purpose.

## Materials and reagents

Highly pure (> 90%) reference standards of PhACs were purchased from Sigma-Aldrich (St. Louis, MO, USA) and Toronto Research Chemicals (Toronto, Ontario, Canada): acetaminophen, acridone, atenolol, bezafibrate, bromazepam, caffeine, carazolol, carbamazepine, chlorpromazine, clarithromycin, codeine, diazepam, diltiazem, erythromycin, fenofibrate, flumequine, fluoxetine, furazolidone, ketamine, ketoprofen, lamotrigine, loratadine, lorazepam, mefenamic acid, mephedrone, methadone, metoprolol, midazolam, nalidixic acid, oxazepam, oxcarbazepine, propyphenazone, salbutamol, sertraline, sotalol, sulfadimethoxine, sulfamethazine, sulfamethoxazole, sulfapyridine, temazepam, trimethoprim, valsartan, valsartan acid, venlafaxine, verapamil, warfarin, zolpidem. The internal standards used as surrogates (IS) were provided by Sigma-Aldrich, CDN Isotopes (Pointe-Claire, Quebec, Canada) and Santa Cruz Biotechnology (Dallas, TX, USA): acetaminophen-d4, bezafibrate-d4, carbamazepine-d10, codeine-d3, diazepam-d5, fenofibrate-d6, lamotrigine-^13^C3, lorazepam-d4, metoprolol-d7, ofloxacin-d3, sulfamethazine-d4, trimethoprim-d3, venlafaxine-d6. Mix of standards used for validation and calibration purpose were prepared by serial dilution starting from a mix of 10 ng/µL in methanol and were stored at −20 °C. Methanol, isopropanol, ammonium acetate (≥98%) and formic acid (puriss p.*a* ≥ 98%) were purchased from Merck (Darmstadt, Germany), and acetonitrile and water HPLC Fisher grade from Fisher Scientific (J.T.Baker, Fisher scientific, Gliwice, Poland). For QTOF-MS/MS calibration purposes, reserpine (*m/z* 609, 28,066) included in the ESI Positive Calibration Solution for the SCIEX X500R System (SCIEX Framingham, MA) was used. The sorbent for lipid removal, QuE Z-Sep/C18, was obtain from Supelco (Darmstadt, Germany). In Table 1, we present the physico-chemical characteristics of the PhAC analyzed including CAS number, molecular formula, molecular and monoisotopical weight, *m/z* for precursor and fragment ion, and the retention time of each analyte.

**Table 1:** Molecular formula, CAS and LC-MS parameters for target PhAC of the validated method.

	PhAC	CAS	Molecular formula	RT(min)^1^	Monoisotopic mass	*m/z* molecular ion [*M* + *H*]^+^	Fragment ion
1	Acetaminophen	103–90–2	C_8_H_9_NO_2_	0.48	151.0633	152.0706	110.0598
2	Acridone	578–95–0	C_13_H_9_NO	4.28	195.0684	196.0757	167.0734
3	Atenolol	29,122–68–7	C_14_H_22_N_2_O_3_	0.38	266.1631	267.1703	145.0638
4	Bezafibrate	41,859–67–0	C_19_H_20_ClNO_4_	7.40	361.1081	362.1153	138.9944
5	Bromazepam	1812–30–2	C_14_H_10_BrN_3_O	0.3	315.0007	316.0080	182.0836
6	Caffeine	58–08–2	C_8_H_10_N_4_O_2_	1.13	194.0804	195.0877	138.0655
7	Carazolol	57,775–29–8	C_18_H_22_N_2_O_2_	3.78	298.1681	299.1754	116.1078
8	Carbamazepine	298–46–4	C_15_H_12_N_2_O	5.31	236.0950	237.1022	194.0949
9	Chlorpromazine	50–53–3	C_17_H_19_ClN_2_S	6.72	318.0957	319.1030	86.0962
10	Clarithromycin	81,103–11–9	C_38_H_69_NO_13_	6.87	747.4769	748.4842	158.1174
11	Codeine	76–57–3	C_18_H_21_NO_3_	0.63	299.1521	300.1594	215.1067
12	Diazepam	439–14–5	C_16_H_13_ClN_2_O	7.3	284.0717	285.0790	154.0413
13	Diltiazem	42,399–41–7	C_22_H_26_N_2_O_4_S	5.53	414.1613	415.1686	178.0305
14	Erythromycin	114–07–8	C_37_H_67_NO_13_	5.78	733.4612	734.4685	158.1176
15	Fenofibrate	49,562–28–9	C_20_H_21_ClO_4_	8.71	360.1128	361.1201	138.9945
16	Flumequine	42,835–25–6	C_14_H_12_FNO_3_	5.22	261.0801	262.0874	244.0775
17	Fluoxetine †	54,910–89–3	C_17_H_18_F_3_NO	6.29	309.1341	310.1414	148.1118
18	Furazolidone	67–45–8	C_8_H_7_N_3_O_5_	1.48	225.0386	226.0459	122.0106
19	Ketamine	6740–88–1	C_13_H_16_ClNO	1.58	237.0920	238.0993	125.0149
20	Ketoprofen	22,071–15–4	C_16_H_14_O_3_	7.05	254.0943	255.1016	105.0328
21	Lamotrigine	84,057–84–1	C_9_H_7_Cl_2_N_5_	1.85	255.0079	256.0152	210.9820
22	Loratadine	79,794–75–5	C_22_H_23_ClN_2_O_2_	7.8	382.1448	383.1521	337.1115
23	Lorazepam	846–49–1	C_15_H_10_Cl_2_N_2_O_2_	6.28	320.0119	321.0183	275.0144
24	Mefenamic acid †	61–68–7	C_15_H_15_NO_2_	7.39	241.1103	242.1176	224.1074
25	Mephedrone	1189,805–46–6	C_11_H_15_NO	1.14	177.1154	178.1227	145.0887
26	Methadone	76–99–3	C_21_H_27_NO	6.32	309.2093	310.2166	105.0328
27	Metoprolol	51,384–51–1	C_15_H_25_NO_3_	2.23	267.1834	268.1907	133.0657
28	Midazolam	59,467–70–8	C18H13ClFN3	4.66	325.0782	326.0855	291.1152
29	Nalidixic acid	389–08–2	C_12_H_12_N_2_O_3_	4.66	232.0848	233.0921	187..0504
30	Oxazepam	604–75–1	C_15_H_11_ClN_2_O_2_	5.89	286.0509	287.0582	241.0528
31	Oxcarbazepine	28,721–07–5	C_15_H_12_N_2_O_2_	4.23	252.0899	253.0972	180.0810
32	Propyphenazone	479–92–5	C_14_H_18_N_2_O	5.26	230.1419	231.1492	189.1024
33	Salbutamol	18,559–94–9	C_13_H_21_NO_3_	0.32	239.1521	240.1594	148.0752
34	Sertraline	79,617–96–2	C_17_H_17_Cl_2_N	6.76	305.0738	306.0811	158.9765
35	Sotalol †	3930–20–9	C_12_H_20_N_2_O_3_S	0.34	272.1195	273.1268	133.0766
36	Sulfadimethoxine	122–11–2	C_12_H_14_N_4_O_4_S	4.01	310.0736	311.0809	108.0443
37	Sulfamethazine	57–68–1	C_12_H_14_N_4_O_2_S	1.58	278.0837	279.0910	92.0500
38	Sulfamethoxazole	723–46–6	C_10_H_11_N_3_O_3_S	2.41	253.0521	254.0594	156.1260
39	Sulfapyridine	144–83–2	C_11_H_11_N_3_O_2_S	0.95	249.0572	250.0645	108.0441
40	Temazepam	846–50–4	C_16_H_13_ClN_2_O_2_	6.7	300.0666	301.0739	255.0679
41	Trimethoprim	738–70–5	C_14_H_18_N_4_O_3_	1.32	290.1379	291.1452	230.1169
42	Valsartan	137,862–53–4	C_24_H_29_N_5_O_3_	7.83	435.2271	436.2344	235.0972
43	Valsartan acid		C_14_H_10_N_4_O_2_	4.24	266.0804	267.0877	206.0602
44	Venlafaxine	93,413–69–5	C_17_H_27_NO_2_	3.32	277.2042	278.2115	58.0656
45	Verapamil	52–53–9	C_27_H_38_N_2_O_4_	6.56	454.2832	455.2905	165.0906
46	Warfarin	81–81–2	C_19_H_16_O_4_	7.61	308.1049	309.1122	251.0695
47	Zolpidem	82,626–48–0	C_19_H_21_N_3_O	3.37	307.1685	308.1758	235.1232

1 : RT: Retention time. †: Validated only with one ion.

## Extraction procedure

For the extraction of PhACs from fish muscles, 0.5 g of fresh sample were placed in an Eppendorf Safe-Lock Tube, 2.0 mL (Eppendorf, Hamburg, Germany) and enriched with 12.5 ng of internal standard, using a solution prepared in methanol at a concentration of 100 ng mL^−1^. The sample was vortexed for 1 min and allowed to stand for 15 min, before adding 1 mL of an acetonitrile/isopropanol mixture acidified with concentrated formic acid (purity ≥98%) at 0.1% V/V; and then vortexed again to homogenize the sample. Subsequently, the sample was exposed to ultrasound for 10 min (Fisherbrand^Ⓡ^ FB15064, Waltham), and then centrifuged for 12 min at 20,817 g at −2 °C (Eppendorf AG centrifuge 5810 R Hamburg, Germany). Once the phases were separated, 0.750 µL of the supernatant were taken in 2-mL vials containing Supel™ QuE Z-Sep/C18, and then vortexed and centrifuged for 6 min under the same conditions described above. Finally, 500 µL of supernatant were transferred into a 2-mL HPLC glass vial and the solvent was evaporated under a gentle stream of nitrogen, and then reconstituted the residual with 500 µL of a mixture (9:1) of ammonium acetate (5 nmol *L* ^−^^1^): acetonitrile. The reconstituted sample was vortexed again for 1 min and analyzed by HPLC-QTOF-HRMS.

**Fig. 1:**
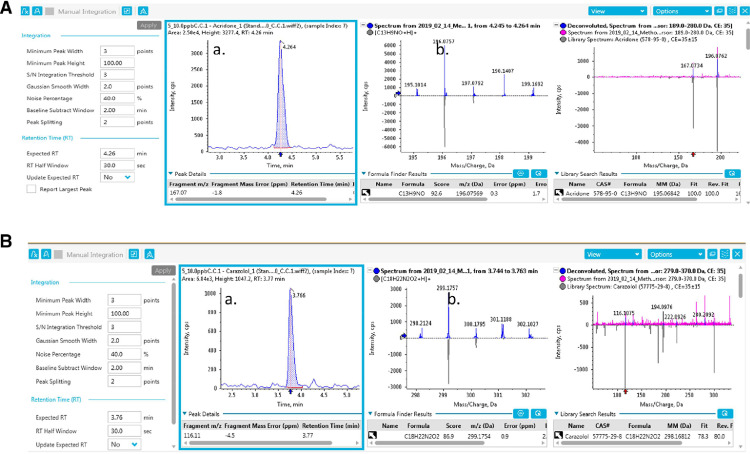
Example of chromatograms for validated PhACs in matrix extracted spiked at 50 ng. mL^-1^.

The chromatographic separation of the analytes was achieved using an UPLC Exion LC AD system (SCIEX, MA) using a thermostated (40 °C) EVO C18 KINETEX packed column (50 mm × 2.1 mm, 2.6 μm, Phenomenex, Torrance, CA). At a flow rate of 0.8 mL min^−1^ the chromatographic separation was carried out in 11 min using as mobile phases 5 mmol *L* ^−^^1^ ammonium acetate + 0.05% V/V formic acid in water (A) and 0.05% V/V formic acid in acetonitrile (B). The injection volume was 10 µL and the autosampler was thermostated at 8 °C. The gradient was as follows: initial conditions (5% B) were maintained for 0.3 min, then was increased to 25% in 5.6 min and then further increased to 40% in the following 1.7 min. Finally, the organic phase increased until 98% in 1.3 min and held for 1.0 min before returning to the initial conditions in 0.1 min, which were maintained for 1.0 min. Fig. 1 shows an example of the chromatographic separation of the validated compounds achieved with this methodology in 11 min. In this case, the sample extract for validation was fortified with the target compounds at a concentration of 50 ng ml^−1^

## Q-TOF-MS/MS-SWATH acquisition

Mass spectrometric analysis was carried out on a hybrid QTOF-MS system X500R, SCIEX (Framingham, MA) equipped with an electrospray ionization (ESI) turboV™ source operated in the positive ion mode. TOF-MS and TOF-MS/MS data were acquired using the SWATH acquisition technology, a data-independent acquisition (DIA) mode that can be applied to fragment any detectable ion from the sample and also collect all MS^2^ [18].

For SWATH acquisition, a single TOF-MS experiment over an *m/z* range from 100 to 1000 was set with an accumulation time of 0.1 s followed by ten MS/MS experiments with controlled Q1 windows widths: *m/z* 100–190, 189–280, 279–370, 369–460, 459–550, 549–640, 639–730, 729–820, 819–910, 909–1000. A collision energy in rampage mode from 20 to 50 eV (35±15 eV) was applied in each mass window, in order to match conditions used to generate the MS/MS library spectra.

With an accumulation time for each MS/MS experiment (window) of 40 ms the total cycle time was 0.588 s. The sprayer probe includes an independent channel for the delivery of a calibration solution (reserpine), that allows to correct any drift in the mass accuracy of the mass analyzer. This calibration was run every 5 samples during the batch analysis. Source conditions were: ion spray voltage: 5500 V; source temperature: 550 °C; nitrogen gas flows (GS1 and GS2): 50 psi; and curtain gas: 35 psi. For qualitative and quantitative data processing Sciex O.S. software V 1.5 (SCIEX) was used.

## Data analysis

**Fig. 2:**
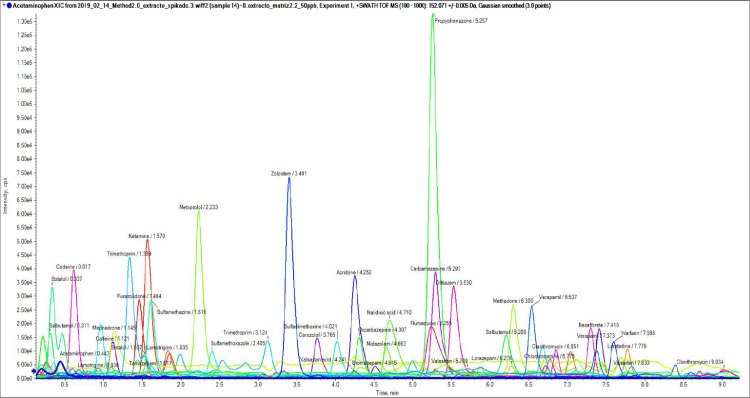
Example of chromatographic peak (a), TOF-MS (b) and Q-TOF-MS/MS (c) for (A) Acridone and B) Carazolol. The MS spectra (b – c) are comparing the acquire MS spectra vs. the database MS spectra. The upper part of the MS spectra corresponds to the experimental acquisition, while the lower part of the MS spectra corresponds to the database library.

The information of the pseudo qualitative and quantitative ions transitions of each target PhAC was imported from the instrumental library database. In this case we used the information of the precursor (using the 1st TOF-MS experiment) and the information of the fragment (using 2–10 TOF MSMS experiments). Therefore, we have the possibility to validate the presence of each PhAC with two ions and to quantify using either the molecular ion and a fragment ion, in case of a substantial fragmentation of the molecular ion in the MS experiment. Table 1 summarizes the molecular ions [*M* + *H*]^+^ and the fragment ions selected for confirmation. Examples of the chromatographic peaks and MS spectra are presented in Fig. 2 for acridone and carazolol. The confirmation of the presence of a target compounds in the sample was carried out taking into account the molecular ions [*M* + *H*]^+^ and the fragment ions, the ion mass error, fragment ion mass error, and retention time.

## Method validation

The selected protocol was validated for specificity, accuracy, precision, linearity, limits of detection (LOD), limit of quantification (LOQ), and matrix effects (ME). The absence of signal above the signal-to-noise ratio (S/N) of 3 at the retention time of the analytes of interest (specificity) in the validation sample was assessed to ensure the quality of the results. The accuracy was determined by spiking the chub fish matrix at three levels of concentration (5, 25 and 50 ng *g* ^−^^1^). Recoveries were calculated as the ratio between the peak area in the extract from spiked fish sample and the peak area in a blank fish extract. The recovery was acceptable between 70 and 130% for majority of compounds, values that fall into the range from other reported studies of pharmaceuticals validated in fish matrix [1,^2^,^19^,20]. The precision of the method expressed by the intra-day repeatability was calculated as the relative standard deviation (RSD%) obtained from the relative recoveries (*n* = 3) for each concentration level, while the inter-day precision was determined by analyzing of the concentration levels for three consecutive days. LOD, defined as the lowest concentration of an analyte that could be distinguished of the matrix signal with a S/N greater than 3, and LOQ, defined as the lowest concentration of a given compound giving a response that could be quantified, with a S/N greater than 10, were estimated from the matrix-matched calibration curves using linear regression [21,22]. Additional to the instrument calibration using the calibration solution, quality control samples were prepared with blank matrix, previously confirmed the absence of the target analytes, with PhACs and internal standards enrichment at concentration of 25 ng mL^−1^, and were injected every 5 samples during the analyses, confirmed with concentration variation lower than 20% with respect to the theoretical concentration. A matrix-matched calibration curve (CC) was prepared by spiking blank chub fish extracts. For quantification purposes the internal standard approach was employed [23,24]. The calibration curve was constructed by linear weighted least-squares regression (1/x as weighting factor). The linearity ranged from 0.5 to 100 ng mL^−1^ corresponding to 1.0 to 200 ng *g* ^−^^1^ f.w. in fish tissue. For all compounds, at least 7 calibration points were considered. ILS was added at constant concentrations (12.5 ng mL^−1^, corresponding to 25 ng *g* ^−^^1^ f.w.). The MEs were calculated as the ratio between the MS peak area spiked into the extract with the peak area in solvent spiked with the same amount. Since fish muscle is a very complex matrix, ME values greater or less than ǀ40ǀ% indicate a strong suppression or improvement of the signal with a consequent impact on the performance of the method. Furthermore, the use of isotopically labelled internal standards helps to compensate for any matrix effect (signal suppression/enhancement) and further improve accuracy and precision. In the present study, twelve isotopically labelled internal standards were used for correction to fulfill the improvements of accuracy and precision. The linear response of the analytes presented a correlation coefficient R^2^ >0.99 for all analytes, excluding mefenamic acid and sulfadimethoxine (R^2^>0.98) where the true values of the calibration curve do not differ more than 20% from the theoretical value. Table 2 shows the results obtained for the validation at 25 ng *g* ^−^^1^ while *Table S1 (supporting information)* compiles the results of the validation at 5 and 50 ng *g* ^−^^1^ fish sample. The validation was performed following the most abundant fragment ion of each analyte. During the evaluation of the recovery, flumequine and nalidixic acid presented the lower values at the 3 concentrations level (50–57% and 44–51% respectively). The recoveries of the 47 compounds were very satisfactory at the levels under study. The signal suppression during the ionization processes of the analytes is strongly marked by the values of the matrix effect, but it is already a well-studied and known phenomenon when performing ESI analyses, since the signal reduction is strongly related to the ionization of the sample in the liquid state before passing to the gaseous phase (in LC-MS) [25], [26], [27], [28], [29] but is independent for each analyte, since it depends on the polarity of the molecule [30]. An example of the matrix effect is the reduction of the linearity of the response of some pharmaceutical compounds such as those mentioned before (mefenamic acid and sulfadimethoxine), which, although an effective cleaning has been developed, seems to continue to disturb the ionization of the compounds. However, the adjusted correlation coefficient is adequate to be able to carry out determinations of these contaminants in such complex matrices as that of fish muscles.

**Table 2:** Method validation parameters at 25 ng PhAC *g* ^−^^1^ fish.

		Intraday performance		Interday performance				
	PhAC	Accuracy (%)	Precision (RSD,%)	Accuracy (%)	Precision (RSD,%)	ME (%)	LOD (ng *g* ^−^ ^1^)	LOQ (ng *g* ^−^ ^1^)
1	Acetaminophen	48	6	40	20	−21	1.7	5.2
2	Acridone	91	3	92	10	−33	0.8	2.3
3	Atenolol	90	7	91	8	−35	1.5	4.5
4	Bezafibrate	85	10	75	21	−25	1.2	3.6
5	Bromazepam	77	5	79	17	−4	0.6	1.7
6	Caffeine	99	6	104	8	54	1.7	5.3
7	Carazolol	97	3	112	16	−22	1.3	4.0
8	Carbamazepine	85	4	85	10	−18	1.5	4.6
9	Chlorpromazine	97	2	96	21	−15	1.3	3.9
10	Clarithromycin	81	5	88	5	−6	1.9	5.8
11	Codeine	86	7	86	17	−5	1.7	5.3
12	Diazepam	94	2	103	13	−55	2.3	6.9
13	Diltiazem	100	3	86	16	−70	0.6	2.0
14	Erythromycin	152	8	133	21	65	1.0	3.1
15	Fenofibrate	153	8	130	11	−83	3.4	10.4
16	Flumequine	57	4	64	20	−88	0.4	1.1
17	Fluoxetine	113	7	101	24	−78	1.9	5.8
18	Furazolidone	102	9	97	20	−23	0.4	1.1
19	Ketamine	80	1	81	12	−24	0.4	1.1
20	Ketoprofen	92	4	86	15	29	1.7	5.0
21	Lamotrigine	77	19	87	19	−45	2.6	8.0
22	Loratadine	113	12	119	18	−69	3.6	10.8
23	Lorazepam	101	3	102	8	−25	1.6	4.9
24	Mefenamic acid	111	4	110	22	−43	1.3	4.1
25	Mephedrone	79	4	84	1	−45	0.3	0.9
26	Methadone	104	14	111	13	−74.5	1.0	3.0
27	Metoprolol	89	11	75	9	14	1.3	3.9
28	Midazolam	94	3	103	21	−41	0.7	2.1
29	Nalidixic acid	51	7	43	15	−39	0.1	0.2
30	Oxazepam	98	3	100	9	−37	0.4	1.4
31	Oxcarbazepine	117	6	114	12	−14	0.4	1.3
32	Propyphenazone	94	3	94	10	−26	0.4	1.3
33	Salbutamol	50	1	53	10	−5	1.0	2.9
34	Sertraline	78	3	83	24	−67	2.8	8.5
35	Sotalol	65	6	68	16	64	1.1	3.4
36	Sulfadimethoxine	125	4	128	20	−45	1.1	3.3
37	Sulfamethazine	172	4	110	9	19	0.8	2.4
38	Sulfamethoxazole	101	8	113	22	−26	0.7	2.2
39	Sulfapyridine	104	5	110	18	28	0.3	1.0
40	Temazepam	100	4	100	9	−20	2.0	5.9
41	Trimethoprim	76	2	80	12	−46	1.8	5.5
42	Valsartan acid	62	21	55	17	48	0.8	2.5
43	Valsartan	78	14	87	12	42	1.0	2.9
44	Venlafaxine	93	5	88	8	−53	1.2	3.7
45	Verapamil	86	4	82	6	−52	3.5	10.7
46	Warfarin	104	1	112	10	−65	1.0	2.9
47	Zolpidem	107	5	106	7	−45	1.0	3.0

The validated method is comparable to other previously reported methods in which PhACs are extracted by USE in the fish muscle (Table 3). In most cases a clean-up with SPE is performed [20,[31], [32], [33], [34]. However, these methods take into account less than 30 PhACs with recoveries ranging from slightly more than 30 to 118%, and the determination of which was developed using low resolution triple quadrupole mass spectrometry (QqQ) instruments. In addition, the matrix effect is reported only in 2 of the 5 methods indicated, with an interval between - 40 and 106%. While the LOQs of these studies show values similar to our proposed method between 0.005 and 61 ng *g* ^−^^1^ of fish. The present approach successfully uses for the first time an alternative cleaning procedure employing zirconia-based sorbents for the analysis of pharmaceutical residues in fish muscle. Previous studies for the determination of PhACs in fish have used PLE with an alternative cleaning phase to the conventional ones consisting of gel permeation chromatography (GPC) [19]. Although this technique may be effective, the use of up to 200 mL of cleaning solvent for each sample makes it an overly expensive method. In only two previous studies, the determination of pharmaceutical residues in fish muscle was performed using high resolution mass spectrometry (HRMS) tools such as Q-Orbitrap and Q-TOF [1,35]. While the former had a large group of contaminants of different types and ME and recoveries varied from −327 to 54% and 40 to 160% respectively, the latter works with freeze-dried fish, less PhAC and its quantification limits range from 1.8 to 303%.

**Table 3:** Characteristics of different validated methods for the determination of PhACs in fish.

Sample weight (g)	Extraction technique	Solvent Volumen (mL)	Purification type	No. compounds validated in other methods	LOD(ng *g* ^−^ ^1^)	LOQ (ng *g* ^−^^1^)	Recovery%	MS instrument	ME (%)	Ref.
0.5 dw*	focused USE	7	SPE	22	0.4–16	N.R.	80 - 118.	QqQ	N.R.	[31]
0.5 dw	USE	10	2 g of alumina + SPE	24	0.01–19 DW	0.04–61 dw	33 - 114	QqQ	N.R.	[20]
1.0	PLE	4 cycles 5 min	GPC	20	0.01–0.4	0.04- 1.4	28 - 126	QqQ	4 - (−86)	[19]
0.5	Homogenization	1	Filtration + frozen	74**	N.R.	0.03 – 5.5	40 - 160	Q-Exactive	54 - (−327)	[35]
1.0 dw	QuEChERS	19	EMR lipid removal	21	0.5– 91 DW	1.8 - 303 dw	10 - 139.	Q-ToF	113 - (−89)	[1]
0.2 dw	vortex	10	filtration	42	0.01 to 2.00	0.1 - 40.2	29 - 188	QqQ	2191 -(−83)	[36]
1	USE	NR	SPE	29	0.01–2.00	0.03 - 6.67	61 - 111	QqQ	NR	[32]
5	USE-McIlvain	15	SPE	27	N.R.	6–30	43 - 103	QqQ	5 - (−40)	[33]
1 z.f.***	USE	0.250	–	9	N.R.	0.005 - 1.5	74 - 100	QqQ	51 - 106	[34]

*dw: dry weight. ** Includes metabolites of PhACs. ***z.f.: zebrafish.

Additionally, our procedure consists of a dispersive SPE clean-up step without affecting considerably the detection and quantification limits obtained during the validation that are in similar ranges between them. Finally, it can be noted that this method was developed using HRMS, which is not the most outstanding feature of the methods for pharmaceutical detection in fish by mass spectrometry, which gives the advantage of being able to make a complete scan of the contaminants and to perform other types of analysis such as suspect screening and non-target analysis, with the advantages of recoveries very suitable, and low limits of detection, even comparable to those obtained with equipment of QqQ that usually are more selective and sensitive. A table with the different characteristics of the above mentioned methods is presented in Table 3.

## Method applicability

Table 4 shows the results of the analysis of fish samples, where the fish are organized according to their species. It should be noted that some fish may have high lipid content such as *Barbus barbus, Onchorhynchus mykiss, Squalius cephalus,* and *Squalius keadicus* which difficults the detection of PhACs without a proper purification of the extract. Verapamil clarithromycin and diltiazem were the most frequently detected compounds in the majority of the fish species analyzed. However, clarithromycin was detected below LOQ in all samples. Verapamil presented concentrations below LOQ in almost all fish samples except in *Squalius cephalus*. In the case of diltiazem, concentrations vary between species ranging from ≤ LOQ to 34 ng.*g* ^−^^1^. Caffeine was detected in six different species in concentrations ranged from ≤LOQ until 69 ng *g* ^−^^1^. Finally, the following PhACs were detected at lower frequency and low concentrations: bezafibrate, carbamazepine, furazolidone, ketoprofen, sulfapyridine, and trimethoprim.

**Table 4:** PhACs detected in European riverine fish.

Sample* (% of lipid content)	Bezafibrate (ng g^-1^)	Caffeine (ng g^-1^)	Carbamazepine (ng g^-1^)	Clarithromycin (ng g^-1^)	Diltiazem (ng g^-1^)	Ketoprofen (ng g^-1^)	Furazolidone (ng g^-1^)	Sulfapyridine (ng g^-1^)	Trimethoprim (ng g^-1^)	Verapamil (ng g^-1^)
*Barbus barbus (7 - 36 %)*	ND	ND	<LOQ - 5.3	<LOQ	<LOQ - 17	ND	ND	ND	ND	<LOQ
*Cottus gobio (10 %)*	ND	ND	ND	<LOQ	11	ND	ND	ND	ND	<LOQ
*Sox Lucius (3 %)*	<LOQ	ND	ND	ND	10	ND	ND	ND	ND	<LOQ
*Onchorhynchus mykiss* *(31 %)*	ND	69	<LOQ	ND	5.7	ND	ND	ND	ND	<LOQ
*Salmo trutta fario* *(4 - 17 %)*	ND	7.8 - 19	<LOQ	<LOQ	<LOQ - 7.2	ND	ND	6.0 - 7.9	<LOQ	<LOQ
*Salmo trutta marmoratus* (4.2 - 5.4 %)	ND	25	ND	<LOQ	3.0 - 7.2	ND	49	ND	ND	<LOQ
*Sander lucioperca (2 %)*	ND	<LOQ	<LOQ	<LOQ	<LOQ	ND	ND	ND	ND	<LOQ
*Squalius cephalus* (4.6 - 27 %)	<LOQ	15 - 32	<LOQ −13	<LOQ	12 - 34	<LOQ	ND	ND	ND	<LOQ - 35
*Squalius keadicus* *(2 - 24 %)*	ND	ND	ND	ND	<LOQ −13	ND	ND	ND	ND	<LOQ
*Thymallus thymallus* (4.7 – 5.2 %)	ND	4.6	ND	<LOQ	3.2 – 6.9	ND	ND	ND	ND	<LOQ

## Declaration of Competing Interest

The authors declare that they have no known competing financial interests or personal relationships that could have appeared to influence the work reported in this paper.

## Appendix Supplementary materials

Image, application 1
