# Supplementary material for: Analysis of pharmaceuticals in fish using ultrasound extraction and dispersive spe clean-up on que Z-Sep/C18 followed by LC-QToF-MS detection
Source: MethodsX. 2020 Jul 26;7:101010. doi: 10.1016/j.mex.2020.101010 (PMC7415924; doi:10.1016/j.mex.2020.101010)
Supplement: Supplementary file 1 [file mmc1.docx]

**Supplementary material**

*Table S1. Validation results at 5 and 25 ng PhAC g^-1^ fish.*

|  |  | **5 ng PhAC g^-1^ fish.** | | | | | **50 ng PhAC g^-1^ fish.** | | | | |
| --- | --- | --- | --- | --- | --- | --- | --- | --- | --- | --- | --- |
|  |  | **Intraday**  **performance** | | | **Interday**  **performance** | | **Intraday**  **performance** | | | **Interday**  **performance** | |
|  | **PhAC** | **Accuracy**  **(%)** | **Precision**  **(RSD, %)** | **ME**  **(%)** | **Accuracy (%)** | **Precision**  **(RSD, %)** | **Accuracy**  **(%)** | **Precision**  **(RSD, %)** | **ME**  **(%)** | **Accuracy (%)** | **Precision**  **(RSD, %)** |
| 1 | Acetaminophen | 77 | 13 | 146 | 81 | 9 | 82 | 2 | 17 | 79 | 10 |
| 2 | Acridone | 78 | 4 | -34 | 104 | 13 | 79 | 7 | -29 | 80 | 5 |
| 3 | Atenolol | 86 | 10 | 9 | 87 | 10 | 84 | 6 | -20 | 77 | 6 |
| 4 | Bezafibrate | 75 | 8 | -32 | 71 | 15 | 82 | 7 | -21 | 74 | 10 |
| 5 | Bromazepam | 92 | 9 | 13 | 88 | 9 | 72 | 10 | -19 | 70 | 7 |
| 6 | Caffeine | 139 | 3 | 45 | 136 | 11 | 103 | 8 | 35 | 93 | 10 |
| 7 | Carazolol | 100 | 14 | -20 | 94 | 11 | 85 | 20 | -28 | 81 | 13 |
| 8 | Carbamazepine | 101 | 5 | -13 | 74 | 3 | 84 | 5 | -21 | 77 | 12 |
| 9 | Chlorpromazine | 99 | 3 | -10 | 72 | 26 | 81 | 4 | -26 | 79 | 21 |
| 10 | Clarithromycin | 74 | 7 | -48 | 86 | 18 | 79 | 19 | -37 | 82 | 8 |
| 11 | Codeine | 131 | 4 | 17 | 107 | 13 | 104 | 10 | 78 | 120 | 8 |
| 12 | Diazepam | 101 | 2 | -65 | 92 | 13 | 89 | 14 | -66 | 96 | 13 |
| 13 | Diltiazem | 119 | 6 | -66 | 80 | 3 | 81 | 23 | -64 | 73 | 10 |
| 14 | Erythromycin | 130 | 2 | 68 | 112 | 8 | 123 | 13 | 60 | 110 | 14 |
| 15 | Fenofibrate | 159 | 19 | -84 | 127 | 22 | 196 | 8 | -86 | 180 | 16 |
| 16 | Flumequine | 51 | 4 | -96 | 61 | 13 | 50 | 12 | -64 | 54 | 11 |
| 17 | Fluoxetine | 109 | 6 | -73 | 90 | 14 | 81 | 15 | -83 | 88 | 21 |
| 18 | Furazolidone | 87 | 2 | -5 | 80 | 13 | 86 | 9 | -1 | 88 | 4 |
| 19 | Ketamine | 88 | 7 | -32 | 91 | 10 | 63 | 10 | -14 | 65 | 4 |
| 20 | Ketoprofen | 79 | 15 | 8 | 59 | 14 | 80 | 2 | 25 | 76 | 6 |
| 21 | Lamotrigine | 89 | 21 | -30 | 72 | 20 | 61 | 18 | -20 | 65 | 12 |
| 22 | Loratadine | 100 | 14 | -62 | 116 | 12 | 99 | 19 | -83 | 103 | 13 |
| 23 | Lorazepam | 99 | 11 | -48 | 104 | 11 | 82 | 3 | -33 | 86 | 7 |
| 24 | Mefenamic acid | 96 | 15 | -49 | 85 | 17 | 95 | 12 | -47 | 99 | 19 |
| 25 | Mephedrone | 71 | 13 | -41 | 87 | 9 | 57 | 17 | -29 | 62 | 8 |
| 26 | Methadone | 92 | 7 | -74 | 99 | 20 | 82 | 20 | -69 | 84 | 12 |
| 27 | Metoprolol | 40 | 47 | 75 | 49 | 25 | 82 | 10 | 23 | 79 | 6 |
| 28 | Midazolam | 90 | 8 | -21 | 85 | 6 | 72 | 15 | -46 | 71 | 18 |
| 29 | Nalidixic acid | 49 | 2 | -37 | 40 | 11 | 44 | 12 | -31 | 39 | 9 |
| 30 | Oxazepam | 88 | 8 | -31 | 80 | 16 | 82 | 3 | -28 | 84 | 7 |
| 31 | Oxcarbazepine | 93 | 8 | -19 | 88 | 6 | 95 | 3 | -3 | 95 | 10 |
| 32 | Propyphenazone | 119 | 4 | -24 | 105 | 8 | 84 | 10 | -16 | 86 | 4 |
| 33 | Salbutamol | 98 | 3 | 0 | 98 | 9 | 51 | 21 | 0 | 54 | 7 |
| 34 | Sertraline | 82 | 4 | -76 | 82 | 21 | 70 | 4 | -80 | 66 | 22 |
| 35 | Sotalol | 79 | 2 | 84 | 89 | 5 | 62 | 7 | 58 | 64 | 7 |
| 36 | Sulfadimethoxine | 108 | 17 | -26 | 91 | 18 | 94 | 6 | -35 | 101 | 18 |
| 37 | Sulfamethazine | 135 | 3 | 24 | 109 | 19 | 107 | 1 | 40 | 100 | 11 |
| 38 | Sulfamethoxazole | 106 | 1 | -12 | 105 | 25 | 101 | 9 | 8 | 102 | 7 |
| 39 | Sulfapyridine | 99 | 7 | 16 | 90 | 6 | 91 | 2 | 61 | 91 | 11 |
| 40 | Temazepam | 88 | 4 | -15 | 96 | 11 | 87 | 5 | -9 | 88 | 4 |
| 41 | Trimethoprim | 113 | 15 | -21 | 91 | 17 | 62 | 14 | -14 | 66 | 7 |
| 42 | Valsartan acid | 63 | 10 | 52 | 55 | 17 | 41 | 4 | 36 | 36 | 9 |
| 43 | Valsartan | 73 | 17 | 24 | 76 | 7 | 86 | 3 | 28 | 79 | 21 |
| 44 | Venlafaxine | 63 | 16 | -21 | 72 | 17 | 76 | 3 | -39 | 70 | 8 |
| 45 | Verapamil | 60 | 3 | 77 | 60 | 10 | 80 | 9 | -55 | 73 | 18 |
| 46 | Warfarin | 112 | 6 | -49 | 104 | 10 | 93 | 12 | -40 | 105 | 10 |
| 47 | Zolpidem | 122 | 6 | -43 | 117 | 12 | 84 | 12 | -30 | 82 | 5 |
